# Overexpression of a SNARE protein AtBS14b alters BR response in Arabidopsis

**DOI:** 10.1186/s40529-014-0055-5

**Published:** 2014-07-12

**Authors:** Zhong Xin Zhu, Hong Bo Ye, Yuan Hu Xuan, Da Nian Yao

**Affiliations:** 1 Agricultural College, Anhui Agricultural University, Changjiangxi Road 130, Hefei, 230036 Anhui China; 2 College of Pharmaceutical Sciences, Wenzhou Medical University, Xueyuanxi Road 82, Wenzhou, 325035 Zhejiang China; 3 Department of Biology, Stanford University, N. Service Road, Stanford, 94305-5020 CA USA

**Keywords:** SNARE, Trafficking, BR, MSBP1, Arabidopsis

## Abstract

**Background:**

N-ethyl-maleimide sensitive factor adaptor protein receptor (SNAREs) domain-containing proteins were known as key players in vesicle-associated membrane fusion. Genetic screening has revealed the function of SNAREs in different aspects of plant biology, but the role of many SNAREs are still unknown. In this study, we have characterized the role of *Arabidopsis* Qc-SNARE protein AtBS14b in brassinosteroids (BRs) signaling pathway.

**Results:**

*AtBS14b* overexpression (*AtBS14b ox*) plants exhibited short hypocotyl and petioles lengths as well as insensitivity to exogenously supplied BR, while *AtBS14b* mutants did not show any visible BR-dependent morphological differences. BR biosynthesis enzyme *BR6OX2* expression was slightly lower in *AtBS14b ox* than in wild type plants. Further BR-mediated repression of *BR6OX2*, *CPD* and *DWF4* was inhibited in *AtBS14b ox* plants. AtBS14b-mCherry fusion protein localized in vesicular compartments surrounding plasma membrane in *N. benthamiana* leaves. In addition, isolation of AtBS14b-interacting BR signaling protein, which localized in plasma membrane, showed that AtBS14b directly interacted with membrane steroid binding protein 1 (MSBP1), but did not interact with BAK1 or BRI1.

**Conclusion:**

These data suggested that Qc-SNARE protein AtBS14b is the first SNARE protein identified that interacts with MSBP1, and the overexpression of *AtBS14b* modulates BR response in *Arabidopsis*.

**Electronic supplementary material:**

The online version of this article (doi:10.1186/s40529-014-0055-5) contains supplementary material, which is available to authorized users.

## Background

Autonomous protein trafficking is one of the major regulatory mechanisms for signal transduction in eukaryotic cells. Membrane trafficking is an intracellular system that can be used to transport proteins; this process includes steps such as budding of transport vesicles in organelles and vesicular fusion to target membrane. It is well known that SAR/ARF GTPases regulate budding processes, and RAB GTPases and soluble N-ethyl-maleimide sensitive factor attachment protein receptors (SNAREs) are key regulators of membrane attaching and fusion. The SNARE proteins are classified into Qa-, Qb-, Qc-SNARE and R-SNARE groups according to their conserved residues within the SNARE motif (Lipka et al. [2007]).

SNAREs are present in the membranes of all subcellular compartments and play a major role in vesicular fusion and membrane biosynthesis. Genome analysis showed that green plants have a larger SNARE family compared with other eukaryotes (Sanderfoot [2007]). Some SNARE proteins are characterized by their function in plants. SYP22/VAM3/SGR3 is a Qa-SNARE that functions in vacuolar and endocytic transport pathways. *syp22* mutants exhibit pleiotropic phenotypes, including wavy leaves, semi-dwarfism, insensitivity to gravity change, and late flowering (Ebine et al. [2012]; Ohtomo et al. [2005]; Shirakawa et al. [2009]; Yano et al. [2003]). R-SNARE protein VAMP727 forms a complex with SYP22, VTI11 and SYP51. This complex plays a crucial role in vacuolar transport, seed maturation, and vacuole biogenesis (Ebine et al. [2008]). Negative dominant form of SYP121, another plant Qa-SNARE, suppresses potassium channel KAT1 and water channel PIP25 traffic to the plasma membrane (Besserer et al. [2012]; Sutter et al. [2006]). Additionally, Qc-SNARE protein AtBS14b has been shown to localize in the Golgi apparatus. (Uemura et al. [2004]).

Brassinosteroids (BRs) are phytohormones that play a crucial role in plant growth and development, including stem elongation, leaf expansion, vascular differentiation, senescence and stress tolerance (Clouse and Sasse [1998]). BRs are recognized by its receptor Brassinosteroid Insensitve 1 (BRI1) and its co-receptor BRI1-Associated receptor Kinase 1 (BAK1), which interacts with BRI1 to enhance BR signaling. Ligand-independent BRI1 trafficking happens between the plasma membrane and the trans-Golgi network/early endosome, followed by degradation in the vacuole (Geldner et al. [2007]). Membrane steroid-binding protein 1 (MSBP1) interacts with and enhances endocytosis of BAK1 to negatively regulate BR signaling (Song et al. [2009]). However, the mechanism and regulatory molecules involved in BRI1, BAK1 and MSBP1 endocytosis are unclear.

Here, we report that AtBS14b, a Qc-SNARE, interacts with MSBP1 in an intracellular compartment. Overexpression of AtBS14b exhibits typical BR defective phenotype including short hypocotyl and petioles in Arabidopsis. Also, *AtBS14b ox* plants are insensitive to exogenously supplied BRs and partially inhibit expression of BR-mediated suppression of BR biosynthesis enzymes. Our results indicate that AtBS14b is the first SNARE protein identified function in BR dependent plant morphogenesis.

## Methods

### Plant materials and growth conditions

The open reading frame (ORF) regions of *AtBS14b* (AT4G14455) and *AtBS14a* (AT3G58170) were recombined into the pABind118 GW binary destination vector via a Gateway LR reaction, and Agrobacterium-mediated transformation into *Arabidopsis thaliana* WS2 ecotype was used to generate *AtBS14b* overexpressing transgenic plants (Bleckmann et al. [2010]). The primers used to clone *AtBS14b* and *AtBS14a* ORF regions are listed in Additional file 1: Table S1. For the transient assay, the ORF region, of *AtBS14b* was recombined into the pEarlyGW 104 binary destination vector. *AtBS14b* insertional mutants, *atbs14b-1* (SALK_016192) and *atbs14b-2* (SALK_124063) were obtained from the SALK line*.* Surface sterilized seeds were sewed on half-strength Murashige and Skoog (MS) medium with 1.2% agar (MS medium) or MS medium supplied with Brassinosteroids (Sigma, Saint Louis, MO, USA). After two days of stratification at 4°C, seedlings were incubated at 22°C in a 24 hours low light chamber (~80 μE/m^2^) for seven days. Seedling plates were photographed and hypocotyl lengths were measured using ImageJ software. For BL treatment experiments, seven days old seedlings were transferred to media containing 1 μM BL and whole seedling were sampled after 3 hours of BL treatment.

### Split GFP assay in tobacco leaves

The N-proximal half of YFP (nYFP) and C-proximal half of CFP (cCFP) sequences were fused to the C-terminal sequences of BAK1, BRI1 or MSBP1 and N-terminal sequences of AtBS14b in PXNGW and PCXGW vectors, respectively. The primers used to clone *BAK1*, *BRI1* and *MSBP1* ORF regions were listed in Additional file 1: Table S1. For transient expression, all binary constructs were introduced into *A. tumefaciens* strains (GV3101). Agrobacterium cells containing split YFP fusion constructs were grown in liquid yeast extract peptone (YEP) medium supplemented with antibiotics (spectinomycin 50 μg/ml and Rifampicin 50 μg/ml). Cultured cells were centrifuged at 4,000 rpm for 5 min at RT, and then the cell pellet was re-suspended in infiltration buffer (10 mM MES, pH 5.6, 10 mM MgCl_2_, 200 μM acetosyringone) after removing supernatant. Cell density was adjusted with infiltration buffer to give an OD_600_ of ~0.5-0.6. Aliquots (0.5 ml) of Agrobacterium cells carrying a split YFP fusion constructs were mixed, and then a syringe was used to infiltrate the mixture into the lower surface of *N. benthamiana* leaves. Plants were incubated in a growth chamber for 36 to 48 hours (Kim et al. [2009]).

### Localization of AtBS14b-mCherry in tobacco leaves

For transient expression of YFP-AtBS14b and YFP-AtBS14a fusion proteins, *AtBS14a* and *AtBS14b* ORFs were cloned into pEarly-GW 104 destination plasmid (Bleckmann et al. [2010]), followed by transient expression in *N. benthamiana* leaves by using the Agrobacterium-mediated transient expression method (Kim et al. [2009]). YFP fluorescence was detected under an Olympus confocal laser-scanning microscope (Fluoview FV 1000, http://www.olympus-global.com/).

### RNA extraction and quantitative RT-PCR analysis

Total cellular RNA was isolated with TRIzol (Takara, Dalian, Liaoning, China) and subsequently treated with DNase (Promega, Madison, WI, USA) to eliminate genomic DNA contamination. For cDNA synthesis, GoScriopt™ Reverse Transcription kit was used follow manufacture’s instruction (Promega, Madison, WI, USA). qRT-PCR analysis used gene specific primers for *BR6OX2*, *CPD*, *DWF4* and *SAUR15* as described Oh et al. [2012]. *Actin2* was used as control (Chen et al. [2012]). All primers used for qRT-PCR were listed in Additional file 1: Table S1.

### Mating-based split ubiquitin assay

For mating-based split ubiquitin assays, MSBP1, BAK1 and BRI1 were cloned into the mating-based split ubiquitin Nub vector pXN25_GW while AtBS14b was cloned into Cub vector pMETYC_GW. Assays were performed as described in a previous study (Lalonde et al. [2010]).

## Results

### Qc-SNARE AtBS14b localized at vesicular compartments

**Figure 1:**
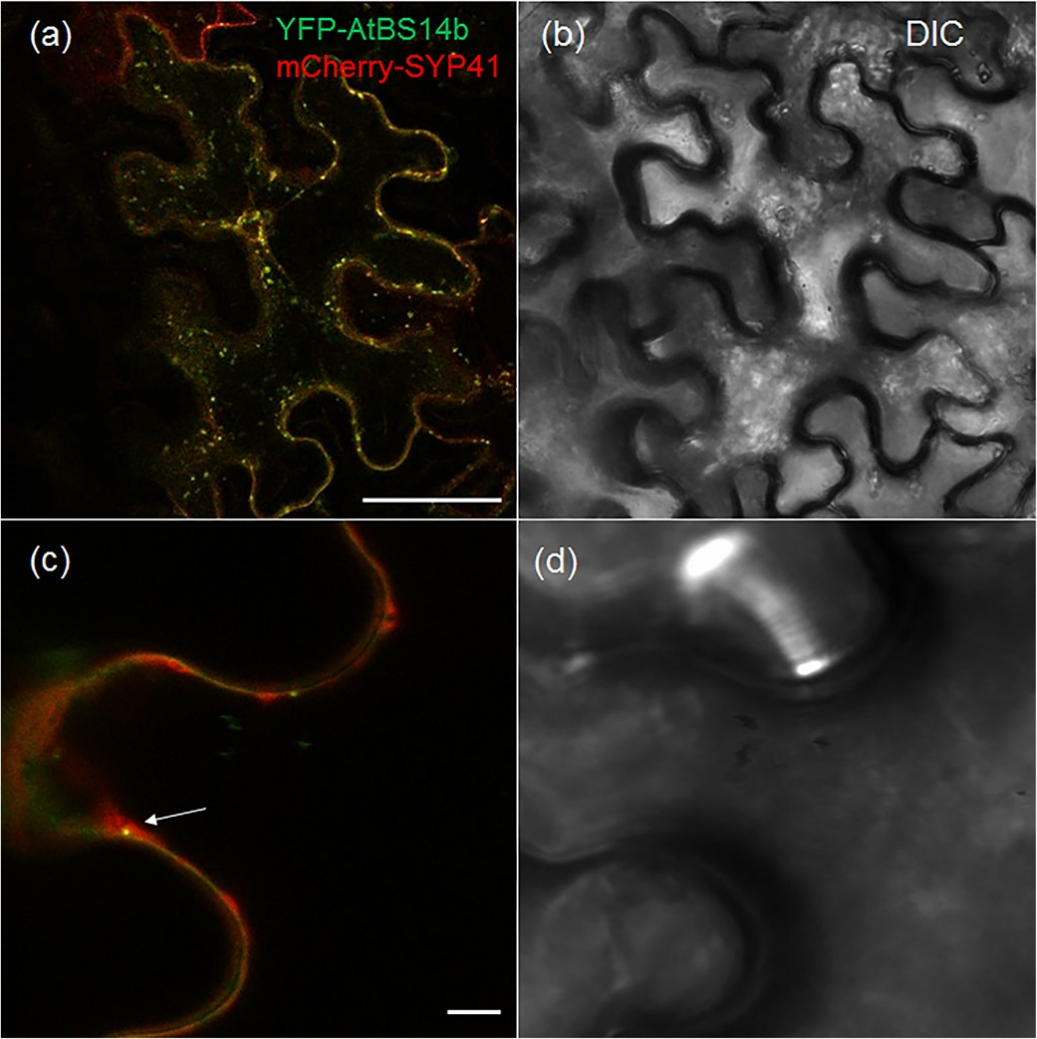
**AtBS14b subcellular localization in** ***N. bethamiana*** **leaves.** YFP-AtBS14b and mCherry-SYP41 proteins expressed in *N. benthamiana* leaves **(a**, **c** fluorescence; **b**, **d** bright field) and arrow heads in **(c)** indicated co-localization of AtBS14b and SYP41. Bars = 20 μm.

SNARE proteins are subdivided into three Q- and one R-SNARE groups. AtBS14a, a Qc-SNARE, was reported to be ubiquitously expressed in all the tissues except pollen and lateral roots (Lipka et al. [2007]). Further, AtBS14b has been analyzed its Golgi localization, but function of AtBS14b in plant growth and development is unknown. To test AtBS14b subcellular localization, ORFs of *AtBS14b* was cloned into pEarly-GW 104 in which yellow fluorescence protein was N-terminally fused to AtBS14b. YFP-AtBS14b fusion protein was subsequently transformed into *N.benthamiana* leaves by using Agrobacterium-mediated transformation. To understand specific localization compartment, tans-Golgi network maker SYP41 was C-terminally fused to mCherry protein. AtBS14b and SYP41 localization were co-localized at vesicular compartments and displayed punctuated patterns surrounding plasma membrane in tobacco leaves (Figure 1).

### *AtBS14b* overexpression plants exhibit short petioles and hypocotyls

**Figure 2:**
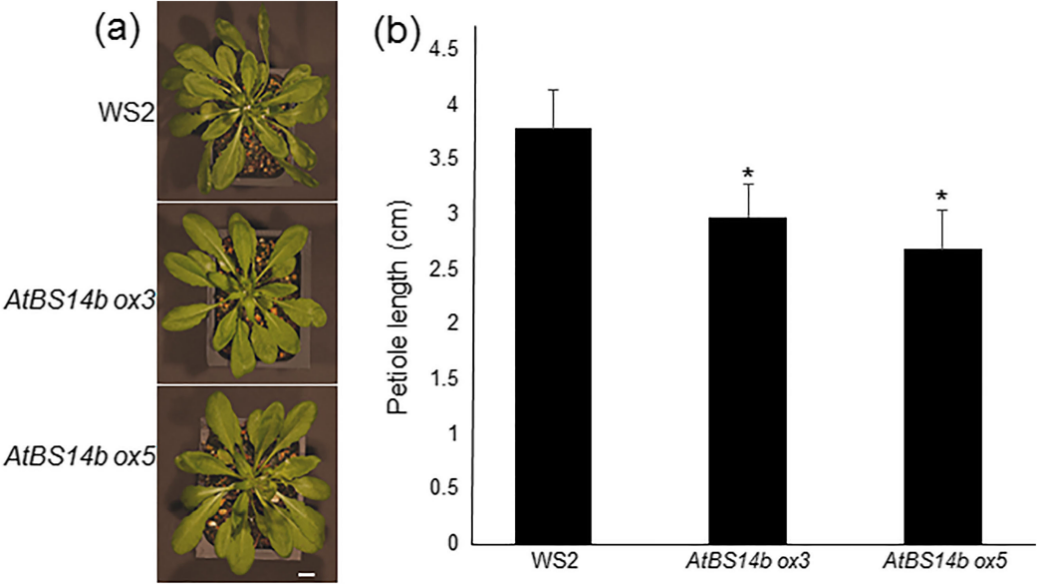
***AtBS14b*** **overexpression phenotype in Arabidopsis. (a)** *AtBS14b* ORF was cloned into pAB118 GW vector and transformed into Arabidopsis WS2 background by using Agrobacteria-mediated transformation. One-month-old *AtBS14b ox3, AtBS14b ox5* and WS2 plants are shown. Bars = 1 cm **(b)** Petiole length from indicated plants shown in **(a)** were measured and the experiments were repeated at least three times with more than 10 plants were analyzed each time (*P < 0.05 t test).

**Figure 3:**
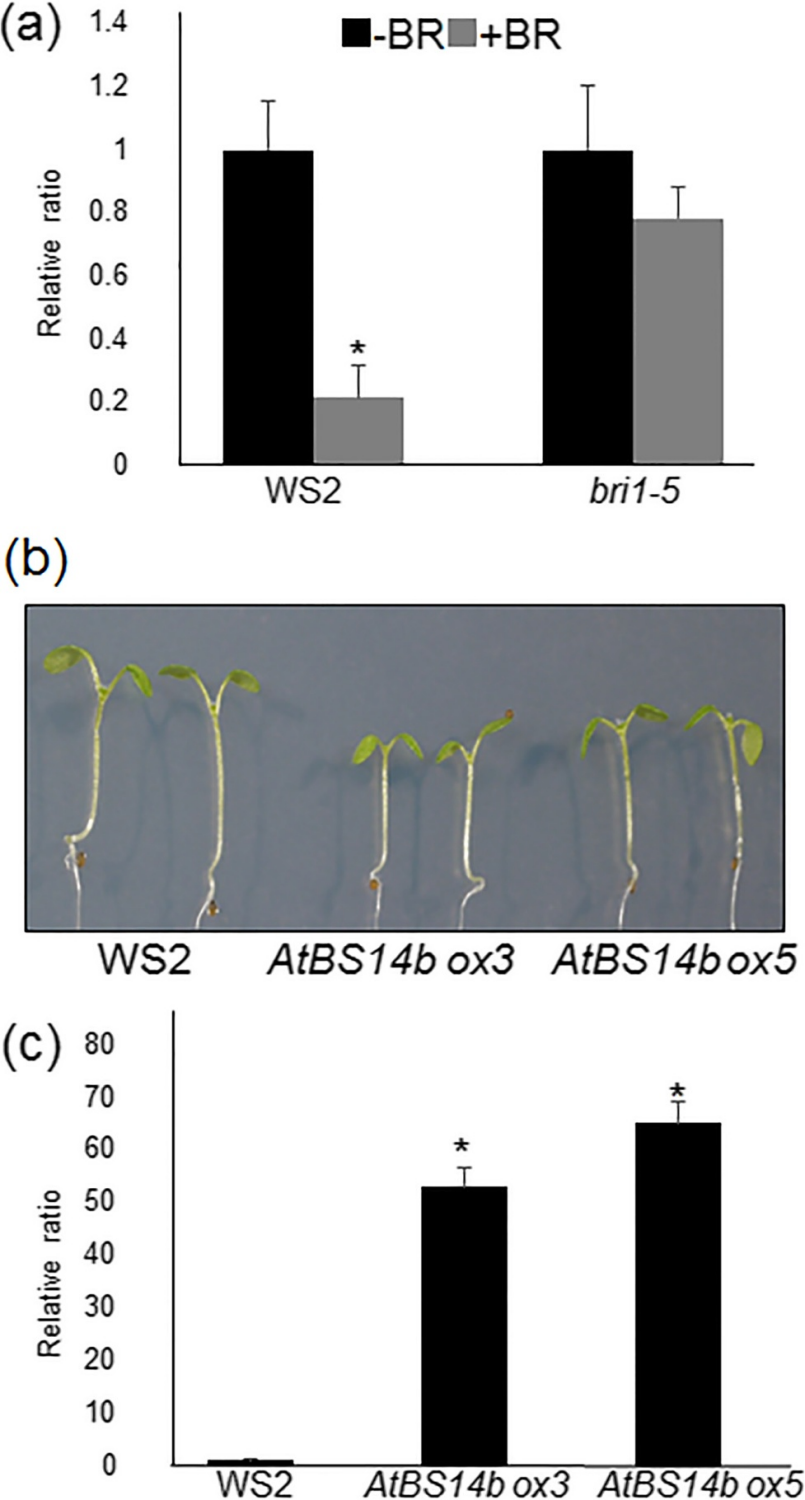
***AtBS14b*** **expression levels and phenotype of** ***AtBS14b*** **overexpression plants. (a)** Seedlings grown on half MS medium for 7 days before treat 1 μM BL for 3 hours. *AtBS14b* expression level was repressed by BL treatment. **(b)** *AtBS14b* overexpression lines (*ox3* and *ox5*) were grown on half MS media under continuous dim light for 7 days, and WS2 and *AtBS14b* overexpression plants were photographed. **(c)** *AtBS14b* expression levels from WS2 and *AtBS14b* overexpression plants were analyzed by qRT-PCR. *Actin* was used as an internal control and the experiments were repeated at least three times with more than 10 plants were analyzed each time (*P < 0.001 t test).

SNAREs are known function in membrane tethering and fusion and play key roles in plant development. To analyze AtBS14a and AtBS14b function, AtBS14a and AtBS14b overexpression (*AtBS14a* and *AtBS14b ox*) plants were generated in WS2 background. More than 15 *AtBS14a ox* and *AtBS14b ox* plants respectively, were successfully selected and further transferred into soil. Short petioles are one of the typical brassinosteroids (BRs) mutant phenotypes. BR mutants also develop short hypocotyls and stem growth (Oh et al. [2012]). *AtBS14b ox* plants developed relatively short petioles in the growth chamber compared to normal leaf length and width (Figure 2), but no obvious differences were observed from *AtBS14a ox* compared to WS2 plants (data not shown). To identify the relationship between BR hormone and AtBS14b, *AtBS14b* expression patterns were analyzed upon BR supplementation. One-week-old seedlings were treated with BR, and *AtBS14b* expression was analyzed by qRT-PCR. The results showed that *AtBS14b* was down-regulated by BR and the suppression requires BRI1 activity (Figure 3a). Hypocotyl growth of *AtBS14b ox* plants was further analyzed in the early seedling stage. One-week-old plants were grown in low, continuous light chamber, and hypocotyl lengths were measured. *AtBS14b ox* plants developed shorter hypocotyls than wild type (Figure 3b) while *AtBS14a ox* plants showed normal hypocotyl growth (Additional file 2: Figure S1a, b). Afterwards, expression levels of *AtBS14b* in overexpression plants were analyzed (Figure 3c). Further, BR-mediated gene expression showed that *AtBS14a* level was not altered by exogenously supplied BR (Additional file 2: Figure S1c). These results indicate that *AtBS14b*, but not *AtBS14a*, is negatively regulated by BR and inhibit hypocotyl and petiole elongation in plants in which *AtBS14b* is overexpressed.

### *AtBS14b ox* plants are insensitive to BL

**Figure 4:**
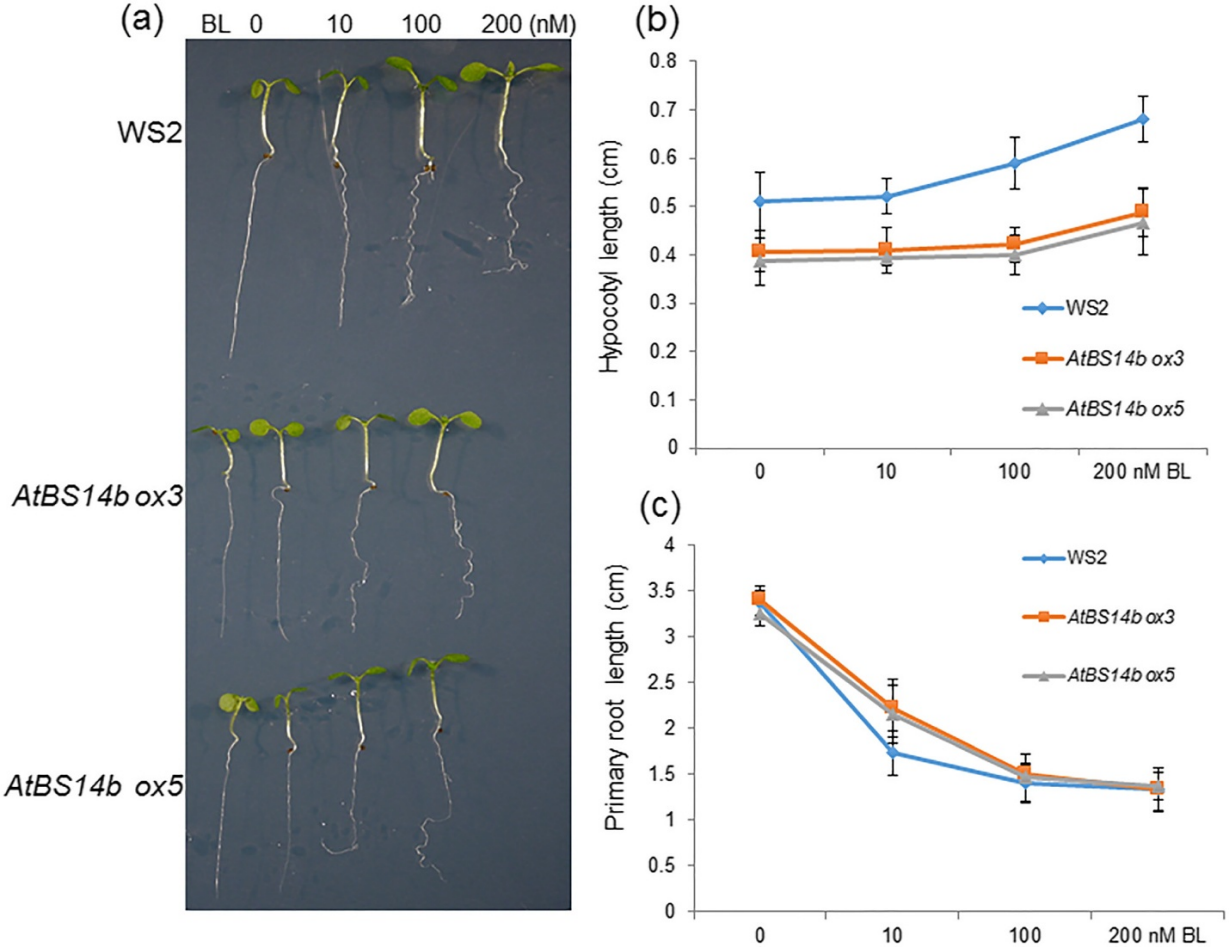
**BR-dependent seedling growth of** ***AtBS14b*** **overexpression plants. (a)** WS2 and two *AtBS14b ox* plants were grown on half MS medium containing indicated concentration of BL under continuous dim light. One-week-old plants were photographed. **(b)** Hypocotyl lengths and **(c)** primary root lengths were measured from one week old seedlings. The experiments were repeated three times.

Brassinosteriods are essential hormones in plants, promoting stem and hypocotyl elongation. To test BR sensitivity of *AtBS14b ox* plants, plants were germinated on MS medium containing BL (0, 10, 100 and 200 nM), and grown for 7 days. Hypocotyl and primary root lengths were measured with ImageJ software. Results show that BR-mediated hypocotyl elongation was partially inhibited in *AtBS14b* ox plants (Figure 4a, b). Also, high concentration of BR was shown to inhibit root growth along with root coiling. Primary root growth patterns indicated that *AtBS14b ox* plants are insensitive to BR especially since drastic differences were observed in 10 nM BR containing medium (Figure 4c). In addition, effects of high concentration of BL (1 μM) on *AtBS14b ox* hypocotyl growth were tested. The data showed that high concentration of BL inhibited hypocotyl growth of wild-type plants, but it promoted hypocotyl length of *AtBS14b ox* plants (Additional file 3: Figure S2a, b). To test effects of other hormones on *AtBS14b ox* hypocotyl growth, gibberellic acid (GA)-dependent hypocotyl growth was analyzed. The data showed that GA changed hypocotyl length of both WS2 and *AtBS14b ox* plants, but no obvious differences were observed between WS2 and *AtBS14b ox* plants (Additional file 3: Figure S2c, d).

Since *AtBS14b* overexpression affected normal plant growth and normal BR response, we further tested *AtBS14b* knock-out mutants. Two independent T-DNA lines were isolated from SALK and named *atbs14b-1* and *atbs14b-2*. T-DNA was inserted in the promoter and first exon in *atbs14b-1* and *atbs14-2*, respectively (Additional file 4: Figure S3). *AtBS14b* expression level was analyzed, and no transcription was detected in two mutant plants (Additional file 4: Figure S3). As shown in Figure 3B, *AtBS14b ox* plants developed shorter hypocotyls than wild type, so hypocotyl growth of *AtBS14b* mutants was further analyzed. As shown in Additional file 4: Figure S3, two mutant lines did not show differences in hypocotyl elongation. In addition, BR dependent primary root growth was analyzed in BL containing media, but no any differences were observed (Additional file 5: Figure S4), suggesting that functional redundancy of SNAREs is believed to occur in plant genomes.

### BR-mediated suppression of three biosynthesis enzyme expressions was inhibited in *AtBS14b ox* plants

**Figure 5:**
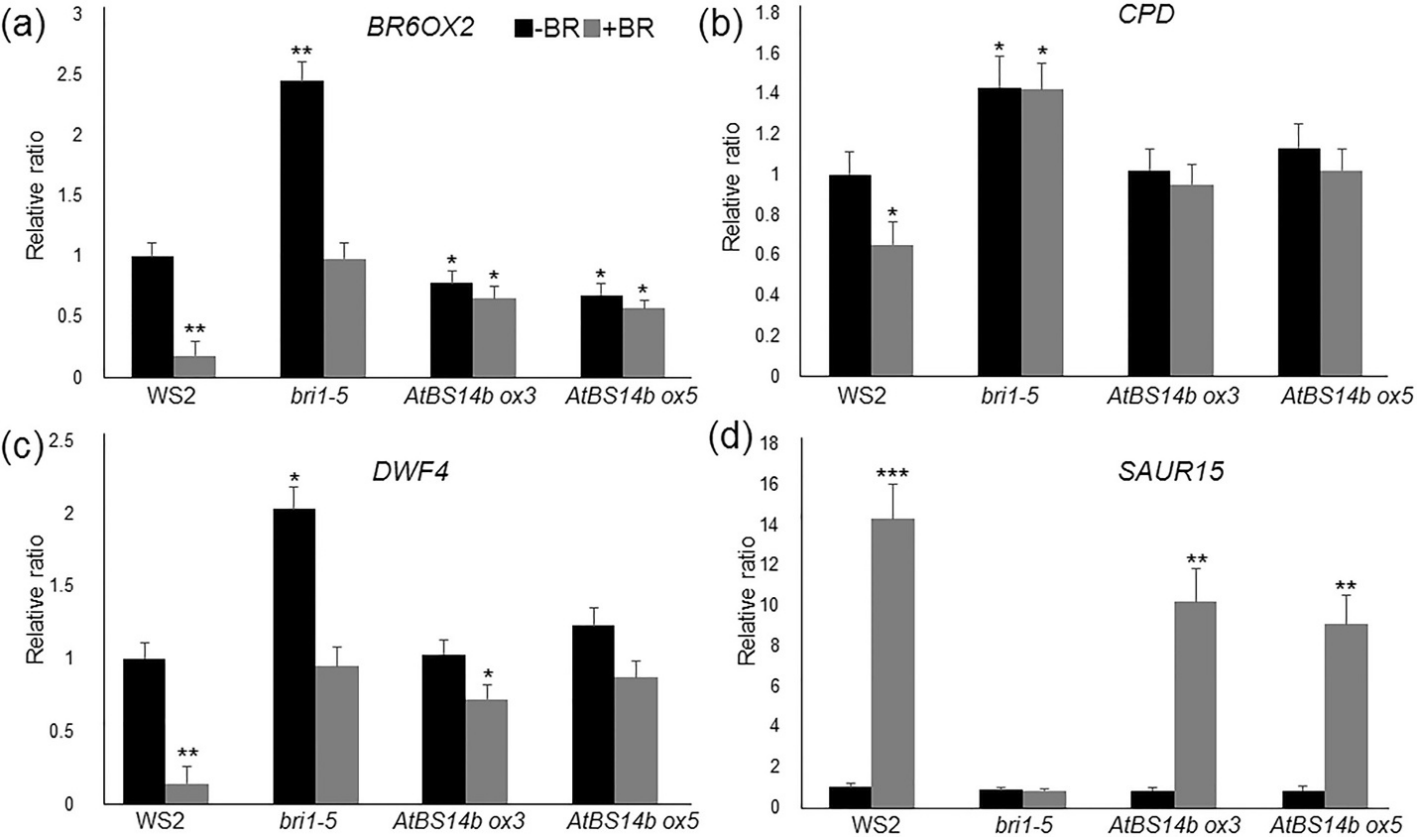
**Expression patterns of** ***BR6OX2*** **,** ***CPD, DWF4*** **and** ***SAUR15*** **in** ***bri1-5*** **and** ***AtBS14b ox*** **plants with or without BL treatment.** Seedlings were grown for 7 days before treatment with 1 μM BL for 3 hours. Black bars indicate plants without BR treatment and gray bars indicate treated with BR for 3 hours. qRT-PCR results show that expression levels of *BR6OX2* **(a)**, *CPD* **(b)** and *DWF4* **(c)** are drastically decreased after BL treatment in WS2, while slight repression was observed in *bri1-5* and *AtBS14b ox* plants*. SAUR15* induction kinetics lower in *AtBS14b ox* plants than in WS2 **(d)**. *Actin* was used as a reference gene and the experiments were repeated at least three times with more than 10 plants were analyzed each time (*P < 0.05, **P < 0.01, ***P <0.001 t test).

*AtBS14b ox* plants, but not *AtBS14b* mutants, show BR dependent phenotypes. Therefore, expression levels of BR signaling genes were further analyzed. In one week old WS2, *bri1-5*, a weak allele of *BRI1* mutant, and *AtBS14b ox* plants were treated with BL for 3 hours and subsequently total RNA was extracted from those lines. qRT-PCR results showed that *BR6OX2*, *CPD* and *DWF4* were the three biosynthesis enzymes (Choe et al. [2001]; Nole-Wilson et al. [2010]; Ohnishi et al. [2012]) whose expressions were dramatically repressed after BR treatment in WS2, but the suppression was inhibited in *bri1-5* indicating gene expression relies on BR signaling. Expression of *BR6OX2* was slightly lower in *AtBS14b ox* plants than WS2, and BR-mediated repression of three genes was inhibited in *AtBS14b ox* plants. Further BR-induced *SAUR15* expression was also analyzed. The results showed that BR-mediated induction of *SAUR15* was inhibited in *bri1-5* and slightly lower in *AtBS14b ox* plants compared to wild type (Figure 5). In contrast, BR-dependent expressions of *BR6OX2*, *CPD* and *DWF4* were examined in *atbs14b* knock-out mutants. The data showed that no significant differences between wild-type and mutants were detected (Additional file 6: Figure S5). Since expression levels of BR biosynthetic genes (*BR6OX2*, *CPD* and *DWF4*) were altered in *AtBS14b ox* plants, BR receptor BRI1 levels was further monitored in 7-day-old *AtBS14b ox* and knock-out mutants. As shown in Additional file 7: Figure S6, *BRI1* transcript was higher in *AtBS14b ox* plants than in WS2 while no differences was observed in knock-out mutants compared to wild-type plants. Taken together, BR-mediated expression patterns of *BR6OX2*, *CPD* and *DWF4* were similar to *bri1-5* and *SAUR15* induction was slightly inhibited in *AtBS14b ox* plants, suggesting that AtBS14b might regulate BR signaling rather than BR biosynthesis genes.

### AtBS14b interacts with MSBP1

**Figure 6:**
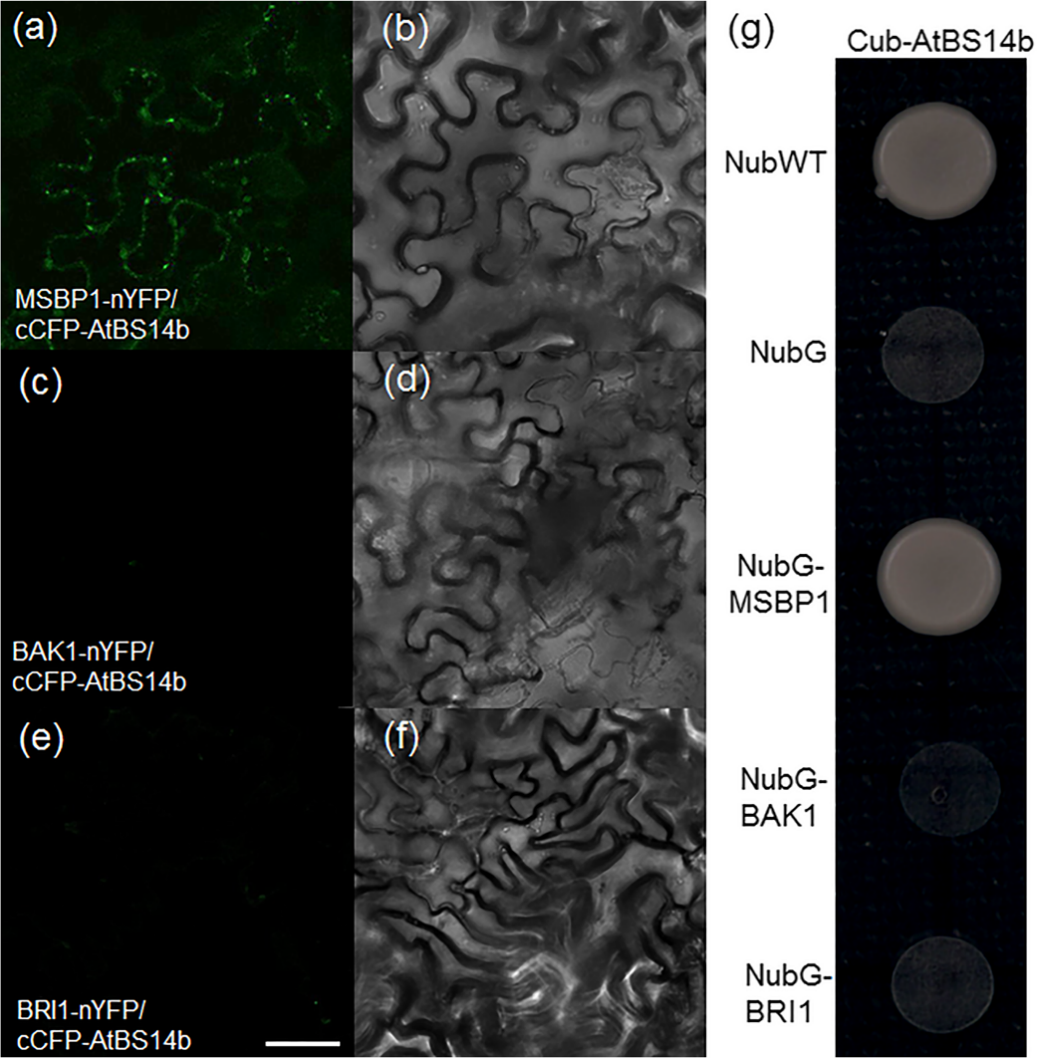
**Interaction between AtBS14b and BRI1, BAK1 or MSBP1 in plants and yeast.** YFP fluorescence and light images were shown. **(a, b)** Reconstitution of YFP fluorescence from MSBP1-nYFP + cCFP-AtBS14b (left fluorescence channel, right brightfield); **(c, d)** BAK1-nYFP + cCFP-AtBS14b; **(e, f)** BRI1-nYFP + cCFP-AtBS14b. Bars = 20 μm. **(g)** Interaction between AtBS14b and MSBP1, BAK1 or BRI1 was analyzed in split-ubiquitin yeast two hybrid system. NubWT and NubG were used as positive and negative control, respectively.

As shown in Figure 1A, YFP-AtBS14b localized at vesicular compartments somehow associated to plasma membrane (Figure 1a), and AtBS14b overexpression inhibited BR signaling. SNAREs are well known as key players that function in membrane tethering and fusion. Therefore, one possibility of AtBS14b regulatory role might directly interact with membrane localized protein involved in BR signaling pathway. To test the possibility, BR receptor BRI1, its co-receptor BAK1 and steroid binding protein MSBP1 were chosen to test direct interaction with AtBS14b. Split GFP system was used to test their interaction in tobacco plants. ORFs of *MSBP1*, *BAK1* and *BRI1* were cloned into PXNGW vector in which N-half of YFP sequences were C-terminally fused to three proteins while ORF of *AtBS14b* was cloned into PCXGW vector in which C-half of CFP sequences were N-terminally fused to AtBS14b. The interaction was tested by co-expressing the two fusion constructs transiently in *N. benthamiana* and by observing YFP fluorescence using confocal microscopy. Fluorescence was observed for co-expression of AtBS14b and MSBP1, but not of BAK1 + AtBS14b or BRI1 + AtBS14b (Figure 6). AtBS14b and MSBP1 interaction was mainly observed at vesicular compartments (Figure 6). To confirm AtBS14b-MSBP1 interaction, we performed a mating-based split-ubiquitin assay. *MSBP1*, *BAK1* and *BRI1* were cloned into the Nub vector pXN25_GW and AtBS14b was cloned into the Cub vector pMETYC_GW. Yeast growth assay showed that AtBS14b interacts with MSBP1 but not BAK1 and BRI1. These results are consistent with split-GFP assay. These data support the hypothesis that AtBS14b directly interact with MSBP1 to modulate BR-mediated plant growth and development.

## Discussion

### AtBS14b is a Qc-SNARE involved in BR signaling

AtBS14b is identified as a Qc-SNARE protein (Lipka et al. [2007]). Our results showed that *AtBS14b* mRNA was repressed upon BR application (Figure 3A), and *AtBS14b* overexpression plants exhibited BR insensitive phenotype as well as inhibit hypocotyl and petiole elongation (Figures 2, 3 and 4). However, overexpression of AtBS14b homologous gene AtBS14a did not change hypocotyl and petiole length, suggesting different function of two BET/SFT family proteins. In contrast, GA application did not show obvious difference on *AtBS14b ox* hypocotyl growth compared to wild-type plants. These results imply that BR may require suppression of *AtBS14b* expression to activate downstream genes involved in BR signaling. VAM3/SYP22 was reported to regulate auxin distribution in leaf vasculature (Shirakawa et al. [2009]), but regulatory role of SNARE proteins in other phytohormones has not been described. AtBS14b is the first SNARE protein identified that functions in BR signaling. *AtBS14b* knock-out mutants were also analyzed for BR dependent phenotypic expression, but no visible phenotypes were observed. SNAREs function as a complex which usually includes three Q- and one R-SNARE proteins, and more than 10 Qc-SNAREs were annotated in Arabidopsis genome. Most Qc-SNAREs are ubiquitously expressed in Arabidopsis (Ebine et al. [2008]; Lipka et al. [2007]). This evidence suggests there may be functionally similar genes occurring; therefore, further genetic screening is required to isolate redundant genes of *AtBS14b* to clarify regulation of AtBS14b to BR signaling pathway. Mutation of a Qa-SNARE SYP22 caused increased tolerance to salt stress (Hamaji et al. [2009]), but *AtBS14b ox* plants did not show differences under salt stress condition (Additional file 8: Figure S7). No other developmental phenotype was observed compared with wild-type plants.

### AtBS14b-MSBP1 interaction may play important role in regulating BR signal pathway

MSBP1 was identified to interact with extracellular domain of BAK1. In addition, the interaction triggered BAK1 endocytosis, inhibiting BR signaling (Song et al. [2009]). MSBP1 is localized at both plasma membrane and endosome and is able to bind BR molecules (Song et al. [2009]). We detected YFP-AtBS14b fusion protein localization at vesicular compartments like the Golgi, which is important for protein endocytosis, and AtBS14b-MSBP1 interactions were mainly detected in vesicular compartments in the cells (Figure 6A). Overexpression of MSBP1 inhibited hypocotyl elongation in Arabidopsis (Song et al. [2009]), which is similar to *AtBS14b ox* plants phenotype. With these results we hypothesize that AtBS14b may interact with MSBP1 in the trans-Golgi network and deliver MSBP1 to plasma membrane. It might provide more chances for MSBP1 to interact with extracellular domain of BAK1 and further enhance BAK1 endocytosis. In *AtBS14b ox* plants, *BR6OX2* level was slightly lower than in WS2, but expression levels of *CPD*, *DWF4* and *SAUR15* were not changed, indicating a specific regulation of AtBS14b on BR signaling and biosynthesis. Interestingly, BR dependent suppression of *BR6OX2*, *CPD*, *DWF4* and BR-mediated induction of *SAUR15* were fully or partially inhibited. More cell biology and genetic experiments are required to understand exact regulatory role of AtBS14b in MABP1 membrane trafficking and BR signaling in *Arabidopsis*.

## Conclusions

*AtBS14b* as a SNARE protein, which is negatively regulated by BR. Overexpression of AtBS14b exhibited short petiole and hypocotyl lengths in *Arabidopsis*. Furthermore, *AtBS14b ox* plants were insensitive to exogenously supplied BR, and BR-mediated suppression of biosynthetic genes (*BR6OX2*, *CPD* and *DWF4*) was inhibited. Split GFP and mating based split ubiquitin yeast hybrid assays showed that AtBS14b interacts with MSBP1 but not BRI1 and BAK1, suggesting a possible regulatory model by which AtBS14b regulates MSBP1 endocytosis and BR signaling.

## Additional files

## Electronic supplementary material

Additional file 1: Table S1.: Primer sequences. (DOC 41 KB)

Additional file 2: Figure S1.:*AtBS14a* expression levels and phenotype of overexpression plants. **(a)** *AtBS14a* overexpression lines (*ox1* and *ox2*) were grown on half MS media under continuous dim light for 7 days and WS2 and *AtBS14a* overexpression plants were photographed. **(b)** *AtBS14a* expression levels from WS2 and *AtBS14a* overexpression plants were analyzed by qRT-PCR. *Actin* was used as an internal control and the experiments were repeated at least three times with more than 10 plants were analyzed each time (***P < 0.001 t test). **(c)** Seedlings grown on half MS medium for 7 days before treat 1 μM BL for 3 hours. *AtBS14a* expression level was analyzed by qRT-PCR. (JPEG 2 MB)

Additional file 3: Figure S2.: High concentration of BR- or GA- dependent seedling growth of *AtBS14b* overexpression plants. **(a)** WS2 and two *AtBS14b ox* plants were grown on half MS medium containing 1 μM BL under continuous dim light. One-week-old plants were photographed. **(b)** Hypocotyl growth from the seedlings shown in **(a)** was measured. **(c)** WS2 and two *AtBS14b ox* plants were grown on half MS medium containing indicated concentration of GA under continuous dim light. One-week-old plants were photographed. **(d)** Hypocotyl length from the seedlings shown in **(c)** was measured. The experiments were repeated at least three times with more than 10 plants were analyzed each time (*P < 0.05 t test). (JPEG 2 MB)

Additional file 4: Figure S3.: Genomic structure, expression levels and morphology of *AtBS14b* knock-out mutants. **(a)** Black boxes indicate exon, white boxes indicate UTR regions and gray triangles indicate T-DNA. T-DNA are inserted in the promoter and first exon in *atbs14b-1* and *atbs14b-2*, respectively. Horizontal arrows indicate qRT-PCR primer binding sites. **(b)** RT-PCR result shows that no *AtBS14b* transcript was detected in *atbs14b-1* and *atbs14b-2. Actin* was used as an internal control and the experiments were repeated at least three times (*P < 0.001 t test). **(c)** Col-0 and *AtBS14b* mutants were grown under continuous dim light for 7 days and seedlings were photographed. (TIFF 1 MB)

Additional file 5: Figure S4.: BR dependent primary root growth of *atbs14b-1* and *atbs14b-2*. Plants were grown on medium containing indicated concentration of BL for 7 days and primary root lengths were measured. No obvious differences were observed from *AtBS14b* mutants compared to Col-0 plants. (TIFF 909 KB)

Additional file 6: Figure S5.: Expression patterns of *BR6OX2*, *CPD,DWF4* and *SAUR15* in *atbs14b* knock-out mutants with or without BL treatment. Seedlings were grown for 7 days before treatment with 1 μM BL for 3 hours. qRT-PCR was used to analyze expression levels of *BR6OX2*, *CPD* and *DWF4. Actin* was used as a reference gene and the experiments were repeated three times with more than 10 plants were analyzed each time. (JPEG 1 MB)

Additional file 7: Figure S6.: Expression level of *BRI1* in *AtBS14b ox* and knock-out mutants. *BRI1* expression levels were analyzed from 7-day-old WS2, *AtBS14b ox*, Col-0 and *atbs14b* mutant seedlings. The experiment was repeated three times (*P < 0.05 t test). (TIFF 15 MB)

Additional file 8: Figure S7.: Growth pattern of *AtBS14b ox* plants under salinity condition. **(a)** WS2 and AtBS14b ox plants were grown on half MS medium with or without 75 mM NaCl for 7 days. **(b)** Root length from the plants shown in **(a)** was measured. More than 10 plants were analyzed. (TIFF 17 MB)

Below are the links to the authors’ original submitted files for images.

Authors’ original file for figure 1

Authors’ original file for figure 2

Authors’ original file for figure 3

Authors’ original file for figure 4

Authors’ original file for figure 5

Authors’ original file for figure 6
