# Supplementary material for: Overexpression of a SNARE protein AtBS14b alters BR response in Arabidopsis
Source: Bot Stud. 2014 Jul 12;55:55. doi: 10.1186/s40529-014-0055-5 (PMC5430330; doi:10.1186/s40529-014-0055-5)
Supplement: Supplementary file 1 — Additional file 1: Table S1.: Primer sequences. (DOC 41 KB) [file 40529_2014_9055_MOESM1_ESM.doc]

Table S1. Primer sequences

| Primer | Sequences |
| --- | --- |
| AtBS14b GW F | GGGGACAAGTTTGTACAAAAAAGCAGGCTTCGGTACCATGAACTTTCGAAGGGAG |
| AtBS14b GW R | GGGGACCACTTTGTACAAGAAAGCTGGGTGACTAGTTTACCCTTTGATGTAGTT |
| AtBS14a GW F | GGGGACAAGTTTGTACAAAAAAGCAGGCTTCGGTACCATGAATCCTAGAAGGGAG |
| AtBS14a GW R | GGGGACCACTTTGTACAAGAAAGCTGGGTGACTAGTTTACCGAGTAAGATAGTA |
| AtBS14b RT F | ACTTTCGTACGTTTCTCG |
| AtBS14b RT R | CCACTTTGTCAAGCAAGC |
| AtBS14a RT F | CCTTGGAGGGATTGCAAG |
| AtBS14a RT R | CCACGAGCGTCAGCATTC |
| BRI1 RT F | AGCTACTCACGGGTAAAC |
| BRI1 RT R | CTATTGTACTGAACCCTC |
| BR6OX2 F | ATGGCGGCGATGAAATACAAAGGA |
| BR6OX2 R | TGTTCCTCCATCAATCTTCTTCTC |
| CPD F | TTACCGCAAAGCCATCCAA |
| CPD R | TCATCACCACCACCGTCAAC |
| DWF4 F | TGGCGGTGTACGGTTTAAGAT |
| DWF4 R | TGGCGGTGTACGGTTTAAGAT |
| SAUR15 F | AAGAGGATTCATGGCGGTCTATG |
| SAUR15 R | GTATTGTTAAGCCGCCCATTGG |
| BRI1 GW F | GGGGACAAGTTTGTACAAAAAAGCAGGCTTCGGTACCATGAAGACTTTTTCAAGCTTC |
| BRI1 GW R | GGGGACCACTTTGTACAAGAAAGCTGGGTGACTAGTTTACCTAATTTTCCTTCAGGAACTTC |
| BAK1 GW F | GGGGACAAGTTTGTACAAAAAAGCAGGCTTCGGTACCATGGAACGAAGATTAATGATC |
| BAK1 GW R | GGGGACCACTTTGTACAAGAAAGCTGGGTGACTAGTTTACCTCTTGGACCCGAGGGGTATTC |
| MSBP1 GW F | GGGGACAAGTTTGTACAAAAAAGCAGGCTTCGGTACCATGGCGTTAGAACTATGGCAA |
| MSBP1 GW R | GGGGACCACTTTGTACAAGAAAGCTGGGTGACTAGTTTACCCTCCTCCTTCTTCAACACAGT |
| Actin F | TCCAAGCTGTTCTCTCCTTG |
| Actin R | GAGGGCTGGAACAAGACTTC |
