# Supplementary figures and images for: Overexpression of a SNARE protein AtBS14b alters BR response in Arabidopsis
Source: Bot Stud. 2014 Jul 12;55:55. doi: 10.1186/s40529-014-0055-5 (PMC5430330; doi:10.1186/s40529-014-0055-5)

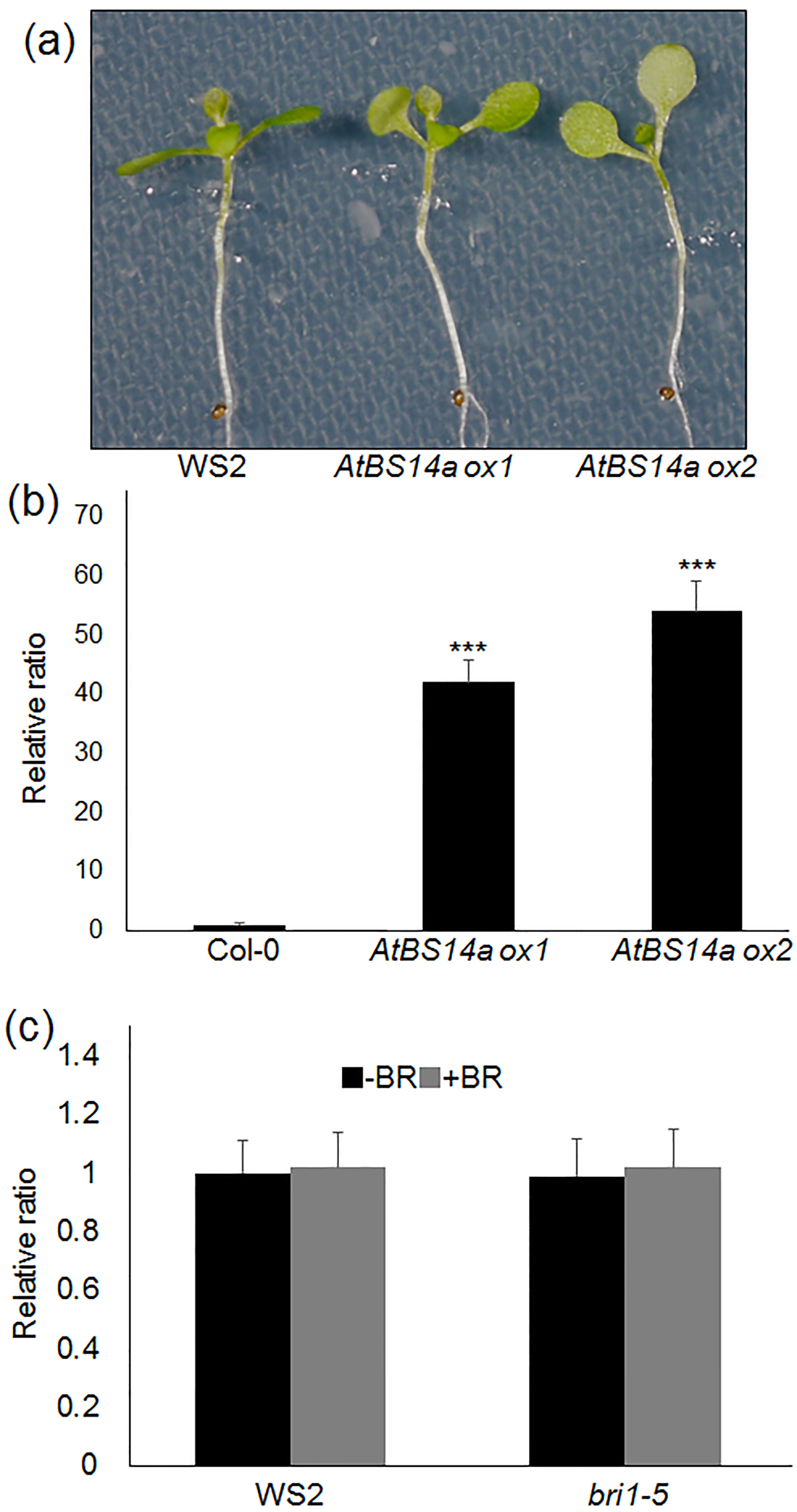

Supplement: Supplementary file 2 — Additional file 2: Figure S1.:AtBS14a expression levels and phenotype of overexpression plants. (a) AtBS14a overexpression lines (ox1 and ox2) were grown on half MS media under continuous dim light for 7 days and WS2 and AtBS14a overexpression plants were photographed. (b) AtBS14a expression levels from WS2 and AtBS14a overexpression plants were analyzed by qRT-PCR. Actin was used as an internal control and the experiments were repeated at least three times with more than 10 plants were analyzed each time (***P < 0.001 t test). (c) Seedlings grown on half MS medium for 7 days before treat 1 μM BL for 3 hours. AtBS14a expression level was analyzed by qRT-PCR. (JPEG 2 MB) [file 40529_2014_9055_MOESM2_ESM.jpeg]

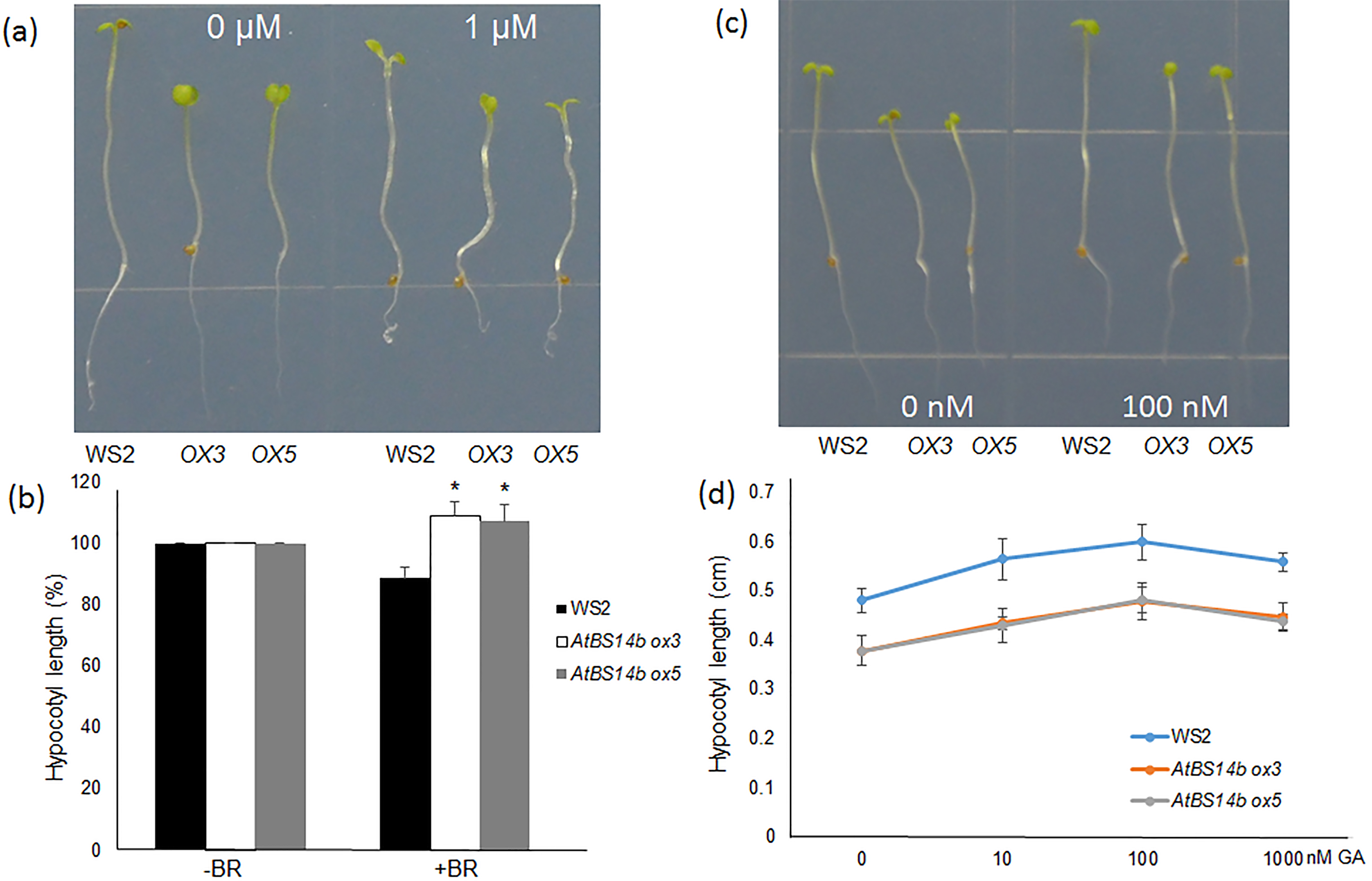

Supplement: Supplementary file 3 — Additional file 3: Figure S2.: High concentration of BR- or GA- dependent seedling growth of AtBS14b overexpression plants. (a) WS2 and two AtBS14b ox plants were grown on half MS medium containing 1 μM BL under continuous dim light. One-week-old plants were photographed. (b) Hypocotyl growth from the seedlings shown in (a) was measured. (c) WS2 and two AtBS14b ox plants were grown on half MS medium containing indicated concentration of GA under continuous dim light. One-week-old plants were photographed. (d) Hypocotyl length from the seedlings shown in (c) was measured. The experiments were repeated at least three times with more than 10 plants were analyzed each time (*P < 0.05 t test). (JPEG 2 MB) [file 40529_2014_9055_MOESM3_ESM.jpeg]

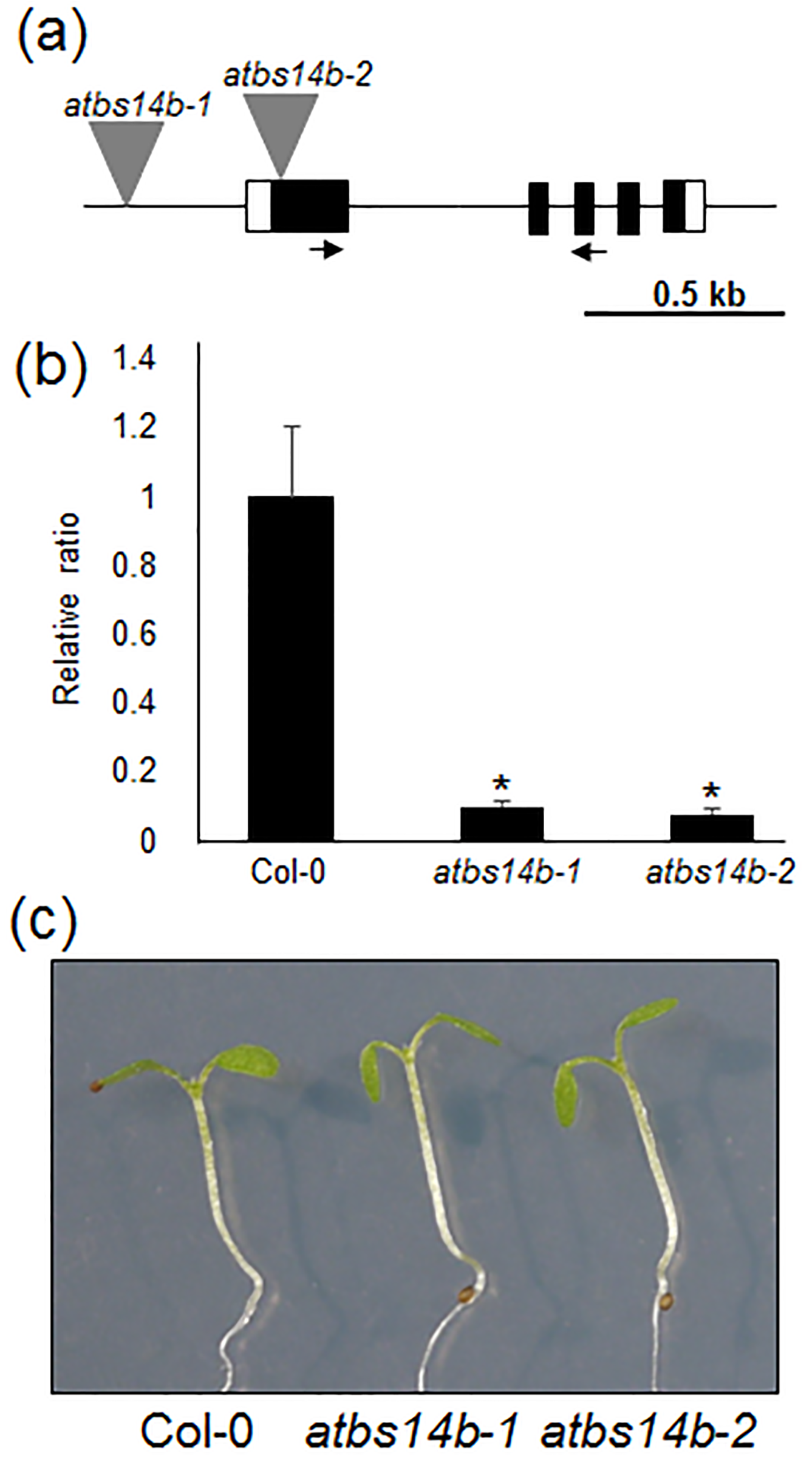

Supplement: Supplementary file 4 — Additional file 4: Figure S3.: Genomic structure, expression levels and morphology of AtBS14b knock-out mutants. (a) Black boxes indicate exon, white boxes indicate UTR regions and gray triangles indicate T-DNA. T-DNA are inserted in the promoter and first exon in atbs14b-1 and atbs14b-2, respectively. Horizontal arrows indicate qRT-PCR primer binding sites. (b) RT-PCR result shows that no AtBS14b transcript was detected in atbs14b-1 and atbs14b-2. Actin was used as an internal control and the experiments were repeated at least three times (*P < 0.001 t test). (c) Col-0 and AtBS14b mutants were grown under continuous dim light for 7 days and seedlings were photographed. (TIFF 1 MB) [file 40529_2014_9055_MOESM4_ESM.tiff]

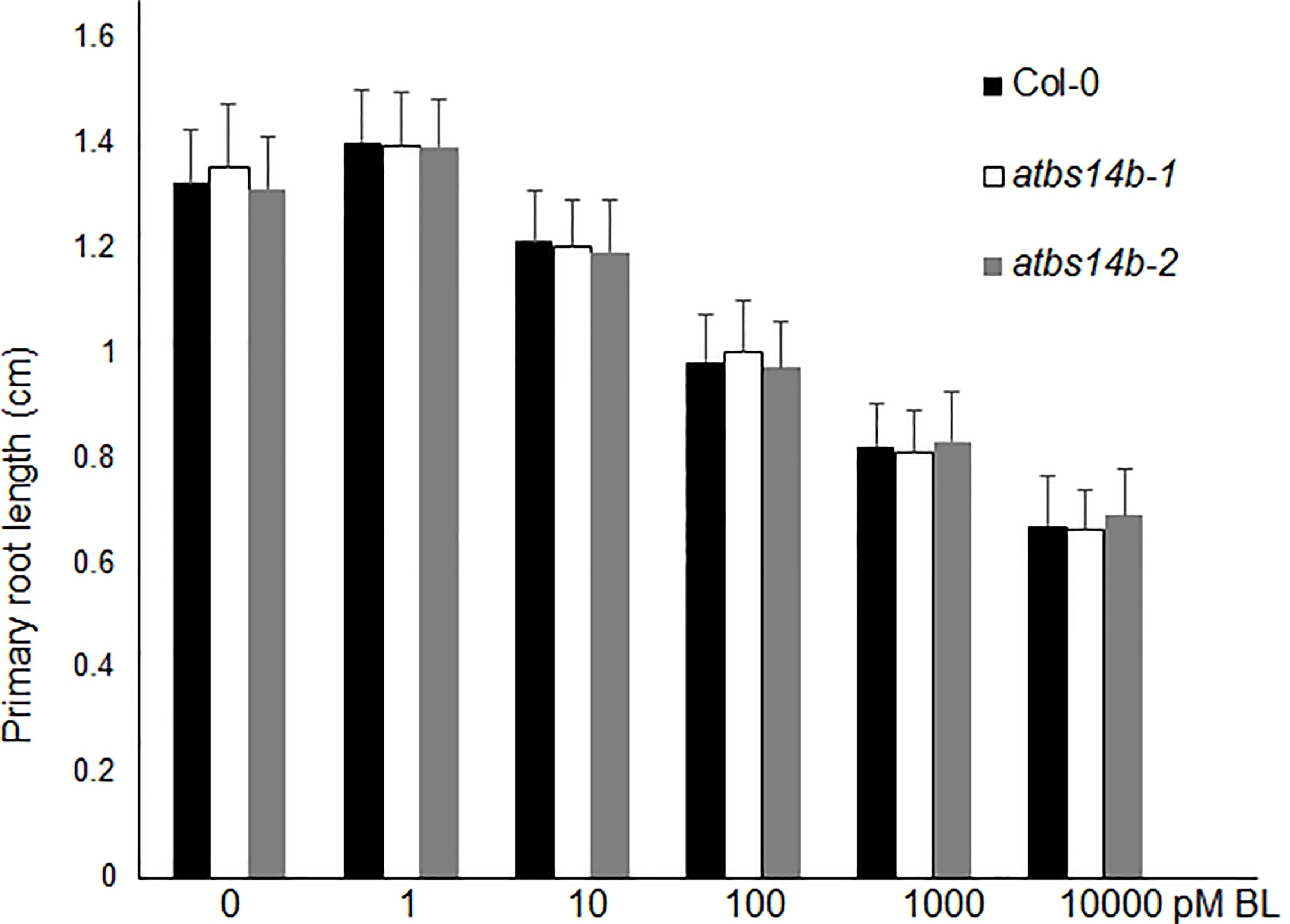

Supplement: Supplementary file 5 — Additional file 5: Figure S4.: BR dependent primary root growth of atbs14b-1 and atbs14b-2. Plants were grown on medium containing indicated concentration of BL for 7 days and primary root lengths were measured. No obvious differences were observed from AtBS14b mutants compared to Col-0 plants. (TIFF 909 KB) [file 40529_2014_9055_MOESM5_ESM.tiff]

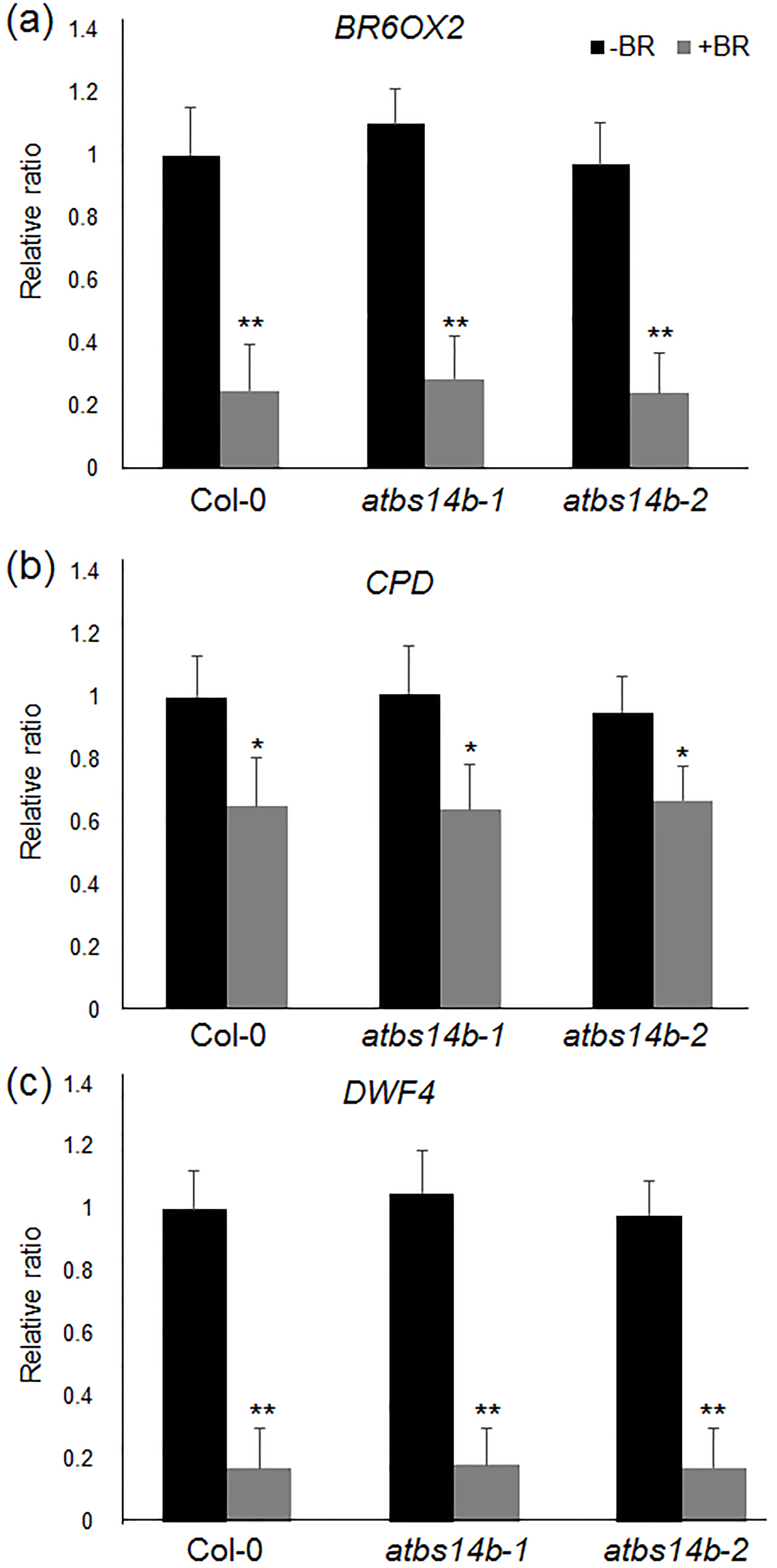

Supplement: Supplementary file 6 — Additional file 6: Figure S5.: Expression patterns of BR6OX2, CPD,DWF4 and SAUR15 in atbs14b knock-out mutants with or without BL treatment. Seedlings were grown for 7 days before treatment with 1 μM BL for 3 hours. qRT-PCR was used to analyze expression levels of BR6OX2, CPD and DWF4. Actin was used as a reference gene and the experiments were repeated three times with more than 10 plants were analyzed each time. (JPEG 1 MB) [file 40529_2014_9055_MOESM6_ESM.jpeg]

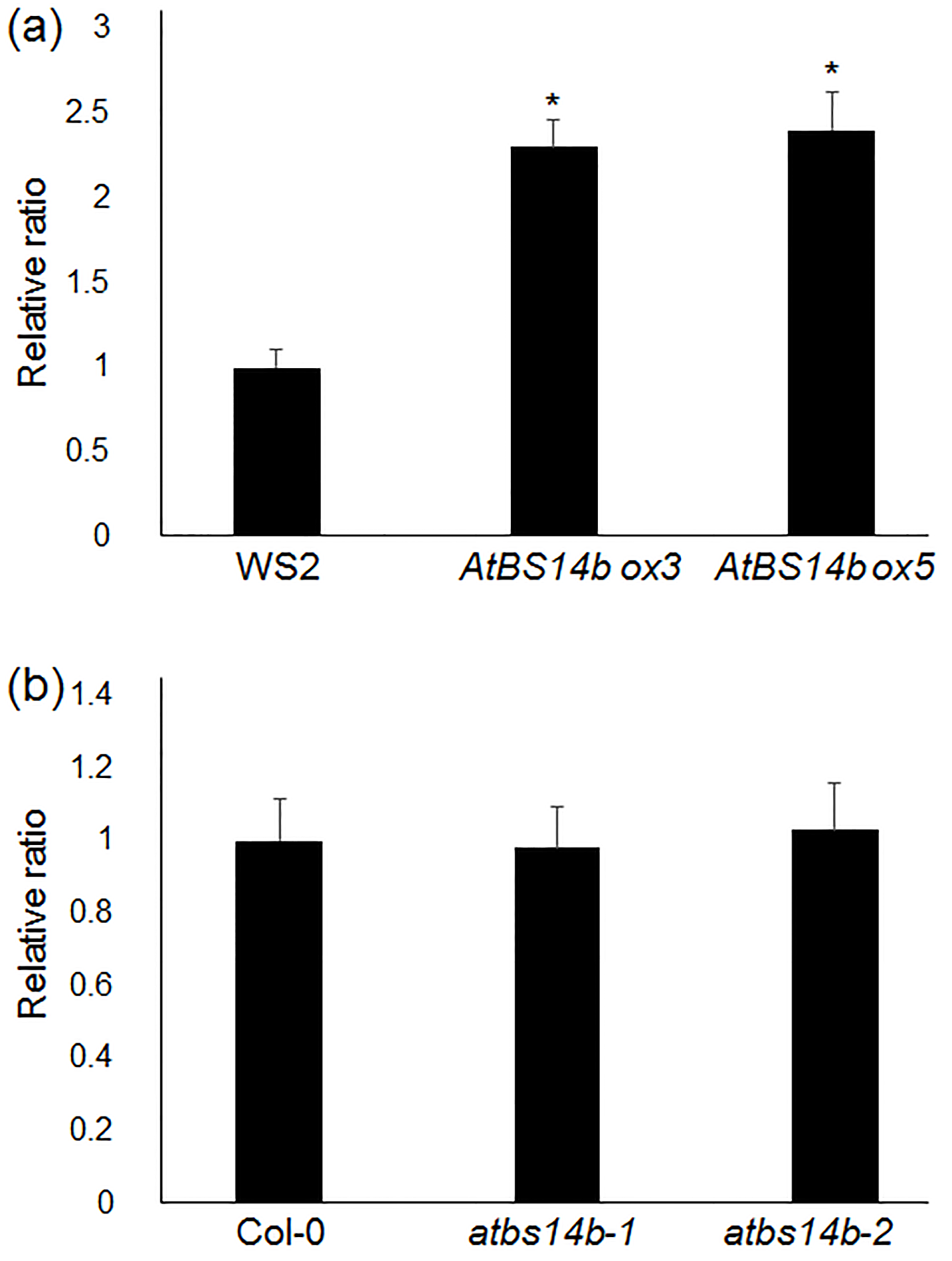

Supplement: Supplementary file 7 — Additional file 7: Figure S6.: Expression level of BRI1 in AtBS14b ox and knock-out mutants. BRI1 expression levels were analyzed from 7-day-old WS2, AtBS14b ox, Col-0 and atbs14b mutant seedlings. The experiment was repeated three times (*P < 0.05 t test). (TIFF 15 MB) [file 40529_2014_9055_MOESM7_ESM.tiff]

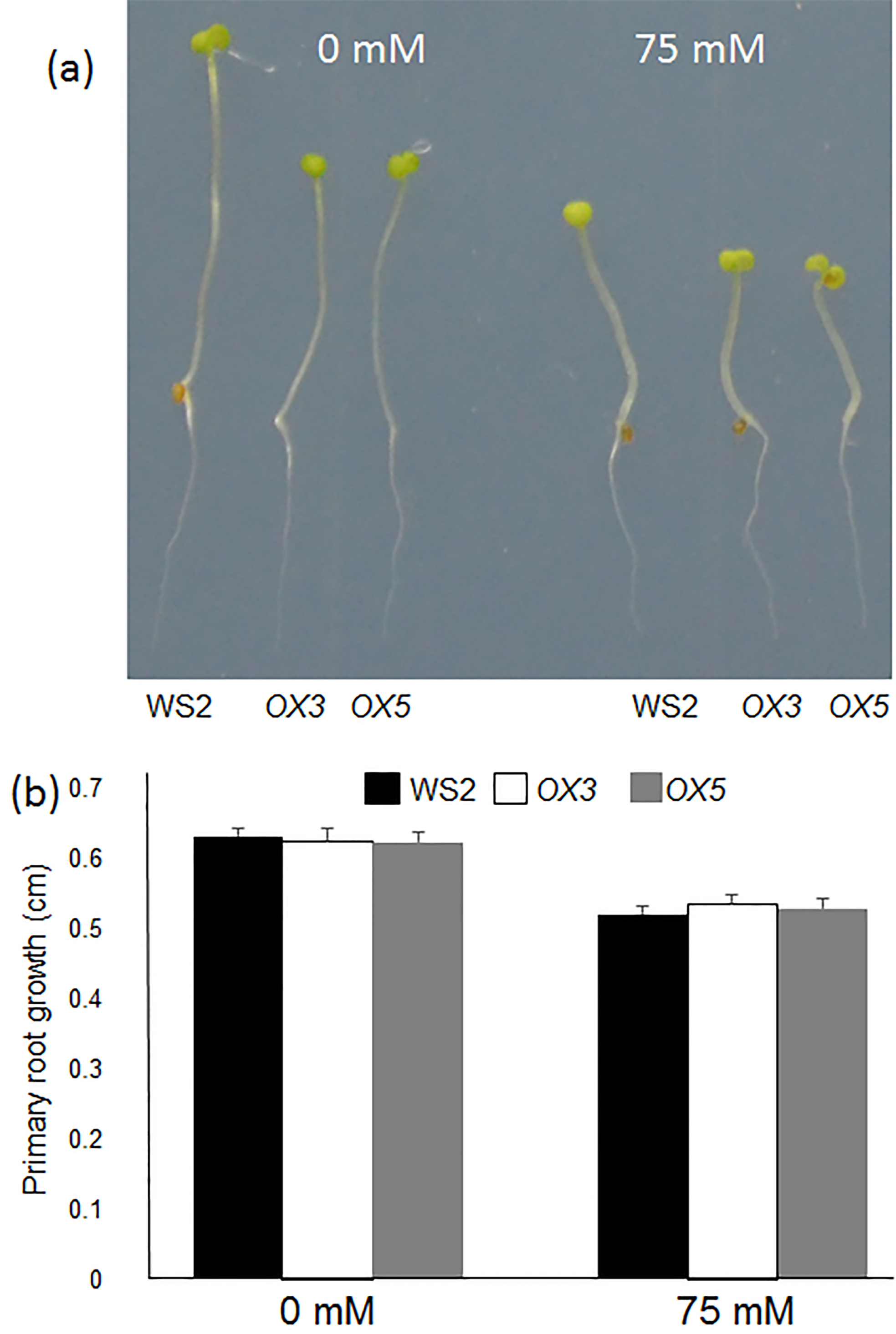

Supplement: Supplementary file 8 — Additional file 8: Figure S7.: Growth pattern of AtBS14b ox plants under salinity condition. (a) WS2 and AtBS14b ox plants were grown on half MS medium with or without 75 mM NaCl for 7 days. (b) Root length from the plants shown in (a) was measured. More than 10 plants were analyzed. (TIFF 17 MB) [file 40529_2014_9055_MOESM8_ESM.tiff]

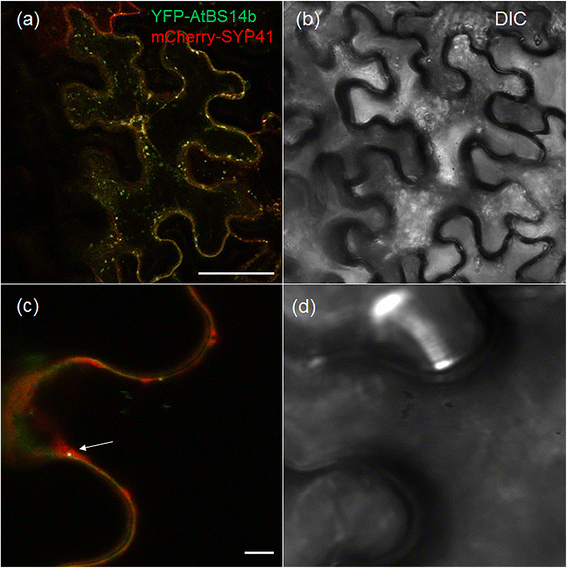

Supplement: Supplementary file 9 — Authors’ original file for figure 1 [file 40529_2014_9055_MOESM9_ESM.gif]

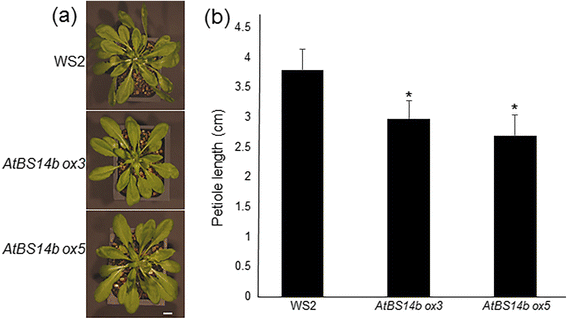

Supplement: Supplementary file 10 — Authors’ original file for figure 2 [file 40529_2014_9055_MOESM10_ESM.gif]

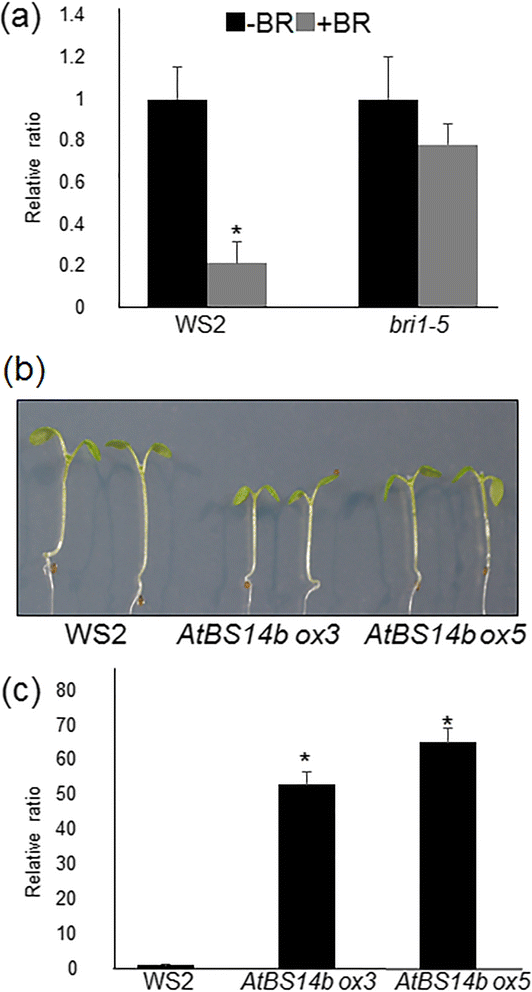

Supplement: Supplementary file 11 — Authors’ original file for figure 3 [file 40529_2014_9055_MOESM11_ESM.gif]

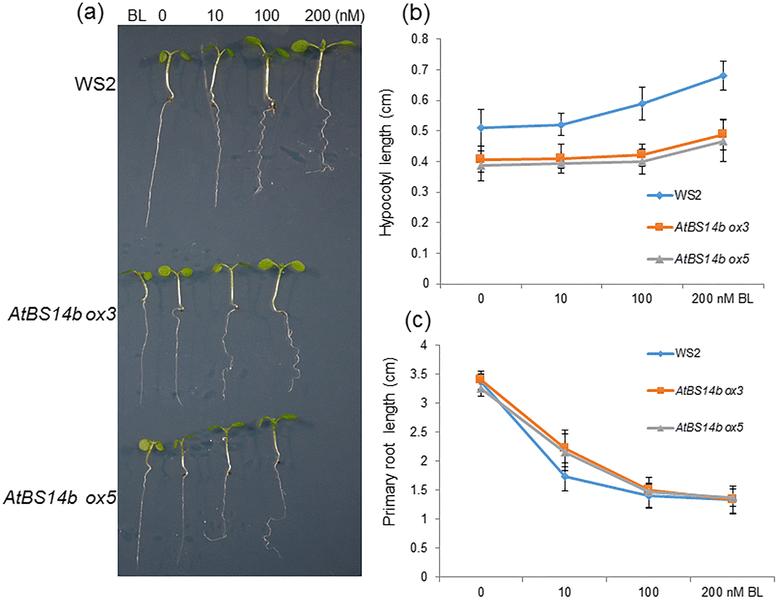

Supplement: Supplementary file 12 — Authors’ original file for figure 4 [file 40529_2014_9055_MOESM12_ESM.gif]

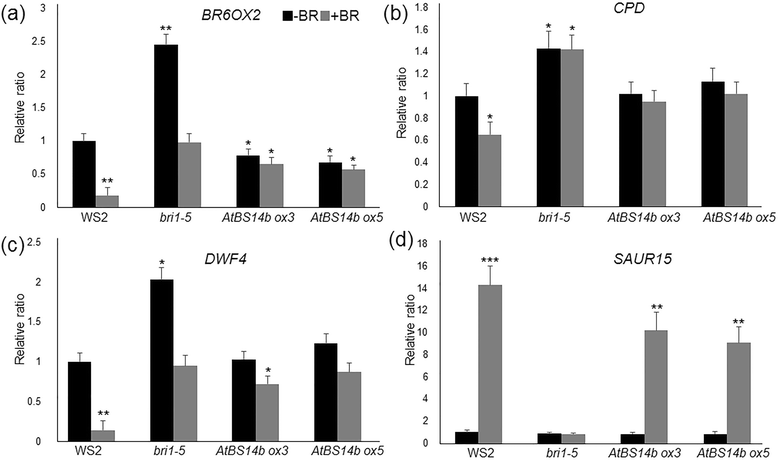

Supplement: Supplementary file 13 — Authors’ original file for figure 5 [file 40529_2014_9055_MOESM13_ESM.gif]

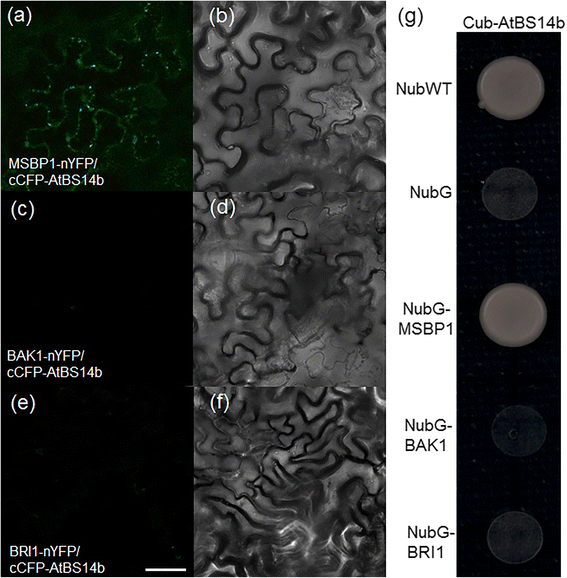

Supplement: Supplementary file 14 — Authors’ original file for figure 6 [file 40529_2014_9055_MOESM14_ESM.gif]
